# A global real-world assessment of the impact on health-related quality of life and work productivity of migraine in patients with insufficient versus good response to triptan medication

**DOI:** 10.1186/s10194-020-01110-9

**Published:** 2020-04-29

**Authors:** Louise Lombard, Mallory Farrar, Wenyu Ye, Yongin Kim, Sarah Cotton, Andrew S. Buchanan, James Jackson, Shivang Joshi

**Affiliations:** 1 Eli Lilly and Company, Lilly Corporate Center, Indianapolis, IN 46285 USA; 2 Neurocrine Biosciences, San Diego, CA USA; 3 Adelphi Real World, Bollington, UK; 4 DENT Neurologic Institute, Amherst, and University of Buffalo School of Pharmacy, Buffalo, New York USA

**Keywords:** Migraine, Health-related quality of life, Work productivity, Triptan, Response

## Abstract

**Background:**

Migraine is a chronic, disabling neurological disease characterized by moderate-to-severe headache pain with other symptoms, including nausea, vomiting, and photophobia. Triptans, while generally effective, are insufficiently efficacious in 30–40% of patients and poorly tolerated by or contraindicated in others. We assessed the impact of insufficient response to triptans on health-related quality of life (HRQoL) and work productivity in patients currently receiving any prescribed triptan formulation as their only acute migraine medication.

**Methods:**

Data were from the 2017 Adelphi Migraine Disease Specific Programme, a cross-sectional survey of primary care physicians, neurologists, and headache specialists and their consulting patients with migraine in the USA, France, Germany, Italy, Spain, and UK. Triptan insufficient responders (TIRs) achieved freedom from headache pain within 2 h of acute treatment in ≤3/5 migraine attacks; triptan responders (TRs) achieved pain freedom within 2 h in ≥4/5 attacks. Multivariable general linear model examined differences between TIRs and TRs in HRQoL and work productivity. Logistic regression identified factors associated with insufficient response to triptans.

**Results:**

The study included 1413 triptan-treated patients (TIRs: *n* = 483, 34.2%; TRs: *n* = 930, 65.8%). TIRs were more likely to be female (76% vs. 70% for TIRs vs TRs, respectively; *p* = 0.011), older (mean age 42.6 vs. 40.5 years; *p* = 0.003), and had more headache days/month (7.0 vs. 4.4; *p* < 0.001). TIRs had significantly more disability, with higher Migraine Disability Scores (MIDAS; 13.2 vs. 7.7; p < 0.001), lower Migraine-specific Quality of Life scores, indicating greater impact (Role Function Restrictive: 62.4 vs. 74.5; Role Function Preventive: 70.0 vs. 82.2; Emotional Function: 67.7 vs. 82.1; all *p* < 0.001), and lower EQ5D utility scores (0.84 vs. 0.91; *p* = 0.001). Work productivity and activity were impaired (absenteeism, 8.6% vs. 5.1% for TIRs vs. TRs; presenteeism, 34.3% vs. 21.0%; work impairment, 37.1% vs. 23.3%; overall activity impairment, 39.8% vs. 25.3%; all *p* < 0.05).

**Conclusion:**

HRQoL and work productivity were significantly impacted in TIRs versus TRs in this real-world analysis of patients with migraine acutely treated with triptans, highlighting the need for more effective treatments for patients with an insufficient triptan response. Further research is needed to establish causal relationships between insufficient response and these outcomes.

## Introduction

Migraine is a chronic, disabling neurological disease characterized by attacks of moderate-to-severe headache pain associated with other symptoms, such as nausea, vomiting, photophobia, and phonophobia [1]. The prevalence of migraine has been estimated at 14% in the USA [2] and 15% in Western Europe [3].

The burden of migraine is considerable. The 2016 analysis of the World Health Organization Global Burden of Disease study reported migraine to be the second highest leading cause of years lived with disability [4], underscoring the widespread prevalence and impact of this condition. An estimated 30-^-^50% of patients experience more than three monthly headache days [5, 6], with these migraine attacks affecting their ability to work, social functioning, and health-related quality of life (HRQoL) [7]. Consequently, effective management of migraine is important in improving HRQoL.

Triptans, i.e. serotonin (5-hydroxytryptamine [5-HT]) agonists with high affinity for 5-HT_1B_ and 5-HT_1D_ receptors, are the most commonly prescribed agents for the acute treatment of migraine [8]. Triptans are recommended as first-line acute treatment of moderate-to-severe migraine attacks [AHS 2019], but persistence tends to be low [9]. Triptans are not effective in all patients; an estimated 30–40% of people with migraine do not respond adequately to triptan therapy in controlled trials [10–13]. Many individuals are intolerant of triptans [11] or are unwilling to take them for reasons that include a fear of adverse events [14]. In addition, triptans have the potential to cause coronary vasoconstriction through activation of 5-HT_1B_ receptors on the vascular smooth muscle [15]; consequently, triptans are contraindicated in people with certain cardiovascular or cerebrovascular conditions such as myocardial infarction, peripheral vascular disease, ischemic heart disease, stroke, transient ischemic attack, and uncontrolled hypertension [16, 17].

The intense pain and other symptoms associated with migraine can have a substantial negative impact on daily life in those experiencing attacks. Moreover, lack of response to acute medication has been linked with an increased risk of progression from episodic to chronic migraine [18]. Patients with migraine report worse subjective wellbeing and reduced HRQoL during attacks and in pain-free periods compared with matched controls [19]. Patients with migraine also report an impact on their ability to work, perform household tasks, and take part in social activities [20]. Suboptimal HRQoL and reduced work productivity are therefore likely to be particularly problematic in patients whose migraine attacks do not respond adequately to triptans; however, few studies have examined outcomes in this patient population.

The objectives of the present study were to determine the HRQoL and work productivity impact on patients with migraine who have an insufficient response to triptan therapy compared with those who have a good response, and to identify factors and characteristics associated with insufficient response to triptans.

## Methods

### Study design

Data were taken from the 2017 (August to December) Adelphi Migraine Disease Specific Programme (DSP), a real-world, point-in-time, cross-sectional survey of primary care physicians, neurologists, and headache specialists and their consulting patients with migraine in the USA, France, Germany, Italy, Spain, and the UK. The DSPs and their validation have been described in detail elsewhere [21, 22].

Physicians completed a patient record form (PRF) for nine consecutive adult patients (aged ≥18 years) with migraine. The PRF contained questions regarding the patient’s demographics and clinical information, including diagnosis, severity of their condition, migraine attack frequency, migraine symptoms, concomitant diagnoses, treatment history, and prescribed migraine medication. An additional question was asked regarding whether the migraine diagnosis was based on International Classification of Headache Disorders criteria, responses to which were Yes, No, or Don’t know. Physicians were compensated for participating in the study according to fair market research rates consistent with the time involved.

Patients for whom a PRF was completed were invited to fill in a patient self-completion form (PSC). The PSC contained questions regarding the patient’s demographics, current symptoms, satisfaction with treatment, and the impact of migraine on their HRQoL (using the Migraine-Specific Quality of Life questionnaire (MSQ; version 2.1 [23];), the EuroQol 5-Dimensions 5-Levels questionnaire (EQ-5D-5 L [24];), the Migraine Disability Assessment questionnaire (MIDAS [25, 26];), and the Work Productivity and Activity Impairment questionnaire:Migraine (WPAI:Migraine [27];). Patients could answer any or none of the questions and were instructed to complete the form without help from their healthcare practitioner.

Patients provided informed consent for use of their anonymized and aggregated data for research and publication in scientific journals by means of a check box on the front page of the PSC. Data were collected in such a way that patients and physicians could not be identified directly. All data were aggregated and de-identified before receipt by Adelphi Real World. DSP data were collected in accordance with Adelphi Real World procedures, which are compliant with the Health Information Technology for Economic and Clinical Health (HITECH) Act and the Health Insurance Portability and Accountability Act (HIPAA). As a matter of routine, Adelphi Real World establishes a Master Services Agreement with each fieldwork partner. This includes the conditions that agencies have to follow local guidelines in addition to adhering to the European Pharmaceutical Market Research Association (EphMRA) Code of Conduct and allows for circumstances (such as in Germany) where local regulations may be different to (more stringent than) EphMRA guidelines. The EphMRA Code of Conduct covers France, Germany, Italy, Spain, UK, and USA. The DSP methodology and questionnaires were reviewed by an official independent body, Freiburger Ethik-Kommission International (FEKI), which granted ethical approval.

### Study population

#### Physicians

Physicians were primary care physicians (PCPs), neurologists, and headache specialists. PCPs and neurologists were required to make treatment decisions for ≥10 and ≥ 20 patients with migraine, respectively, in a typical month.

#### Patients

Patients were included in the analyses if they had a physician-confirmed diagnosis of migraine, had completed a PSC, were currently receiving any formulation of a prescribed triptan as their only acute medication for migraine, and answered the following question, considering their condition over the past 3 months: “In approximately how many migraine attacks would you say your prescribed acute medication stops the pain entirely within 2 hours of taking the medication?”; response options ranged from 0 to 5 of 5 attacks.

Two cohorts of patients were defined: patients with an insufficient response to triptans, hereafter described as triptan insufficient responders (TIRs), and patients with a good response to triptans, hereafter described as triptan responders (TRs). TIRs were those patients who reported achieving pain freedom within 2 h of taking their acute medication in ≤3 of 5 attacks; TRs were those who reported achieving pain freedom within 2 h in ≥4 of 5 attacks This conservative definition of response to triptan medication was based on a synthesis of previous findings [12, 28, 29], with freedom from pain 2 h after taking medication in at least 4 of 5 attacks addressing the need for speed and consistency of pain freedom. The terms TIR and TR were used in the interest of brevity, rather than to place blame on the patient for their lack of response to treatment.

#### Outcomes

The MSQ (version 2.1) measures three aspects of HRQoL: how migraine attacks restrict daily social and work-related activities (Role Function – Restrictive); how migraine attacks prevent these activities (Role Function – Preventive); and the emotional impact of these migraine attacks (Emotional Function) [23]. Response options range from 1 (none of the time) to 6 (all of the time). Computed raw scores were linearly transformed to a 0–100 scale, with 100 indicating full functionality.

The MIDAS questionnaire was designed to measure and quantify migraine-related disability. MIDAS records the number of days reported as missing or with reduced productivity at work or home and social events because of headache [25, 26], with higher scores indicating greater disability. Disability categories are: 0–5 = little or no disability, 6–10 = mild disability, 11–20 = moderate disability, and ≥ 21 = severe disability.

The EQ-5D-5L consists of the EQ-5D-5L descriptive system, which measures mobility, self-care, usual activities, pain/discomfort, and anxiety/depression, and the EQ visual analog scale (VAS) [24]. EQ-5D-5L utility and VAS scores were calculated. The utility score is defined as the numeric index of each patient population’s health state; the VAS score is defined as the average numeric rating of the VAS score, with 0 corresponding to the worst imaginable health state and 100 to the best imaginable health state.

The WPAI:Migraine questionnaire assesses migraine-related work and activity impairment [27]. Four scores are calculated: absenteeism, presenteeism (reduced effectiveness while at work), overall work impairment, and activity impairment. Each score ranges from 0 to 100% after transformation, with higher scores indicating greater impairment.

### Statistical analyses

Numeric variables were described by their frequency, mean, and standard deviation. Categorical variables were described by their frequency and percentage.

TRs and TIRs were compared using a two-sample *t*-test for continuous variables and chi-squared or Fisher’s exact tests for categorical variables.

An adjusted analysis was conducted for HRQoL and work productivity related to migraine as outcome measures: a multivariable general linear model (GLM) was applied to examine differences in disease disability, HRQoL, and work productivity scores between TIRs and TRs. The GLM was fitted using TIRs vs. TRs as a main effect and controlling for age, sex, migraine headache day frequency, comorbidities, duration of illness, preventive medication use, and presence of aura. Adjusted means with standard errors (SEs) were reported.

Backward logistic regression was used to determine factors associated with TIR vs. TR. The candidate continuous covariates included age; MSQ Total Score, MSQ Role Function – Restrictive, Role Function – Preventive, and Emotional Function domain scores; EQ-5D-5L Utility score (cross-walked to 3-level score); physician-reported monthly headache days over the past 3 months; number of migraine-related headache days; and physician-reported headache severity on average over the past 3 months. The candidate categorical covariates included physician specialty; sex; diagnosis of migraine with aura; diagnosis of migraine without aura; days since the patient received a diagnosis of migraine categorized by five levels, four of which were based on quartile distribution of non-missing values and one level for missing values; country; health insurance that includes cover for migraine medication; cardiovascular risk status (mild versus others); comorbidities (depression, anxiety, stress, sleeping disorder, neck pain); whether the patient ever misused opioid medication; diagnosis of menstrual migraine; diagnosis of menstrual-related migraine; symptoms currently experienced: pain on one side of the head; symptoms currently experienced: pain on both sides of the head; symptoms currently experienced: nausea; symptoms currently experienced: vomiting; when acute medication is taken relative to the start of the attack (At the first sign of a migraine [before the pain starts], When the pain starts, or After the pain has started and I have an idea of how severe it is); taking over-the-counter (OTC) medication for migraine attacks; and whether the patient ever received preventive medication for their migraine. A significance level of 0.1 was required for a variable to stay in the model. Age and sex were forced to remain in the model. Odd ratios (ORs), 95% confidence Intervals (CIs), and *p*-values from the final model were reported.

All statistical tests were conducted at a two-sided 5% significance level. Summary statistical information was based on non-missing data. For modeling, all variables had no or lower missing values except for variable duration of disease with 41% missingness. To include more patients in modeling, a categorical variable with 5 levels was created from the duration of disease by quartile on non-missing value and a separate level for missing value to replace the duration of disease in models. For logistic regression model, there were 380 patients (380/1413 = 26.8%) excluded from modeling due to missingness. No patients were excluded from the adjusted analysis of HRQoL and migraine-related work productivity. Analyses were conducted using SAS Enterprise Guide 7.12 (SAS Institute, Cary, NC, USA).

## Results

### Patients and physicians

**Fig. 1:**
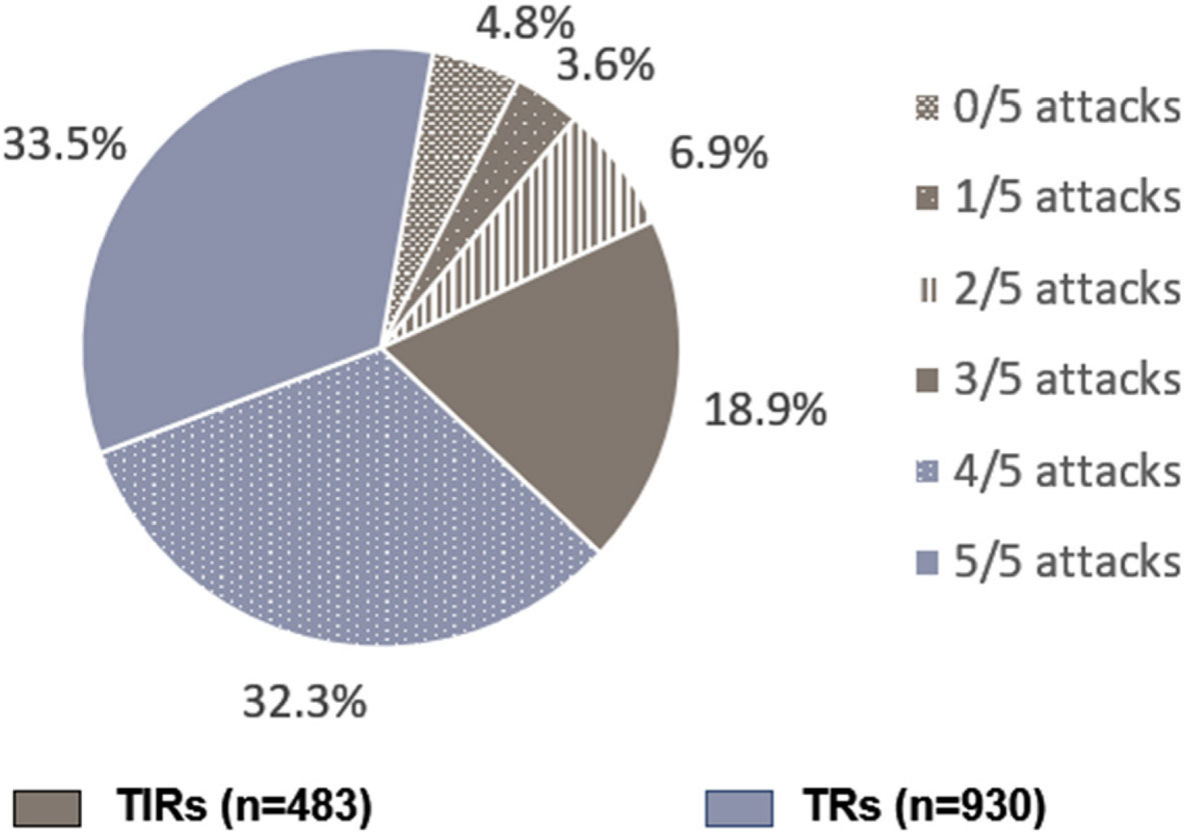
Patient-reported response to triptans: attacks free from pain within 2 h of medication use

The study population included 1413 triptan-treated patients (France, *n* = 195 [14%]; Germany, *n* = 282 [20%]; Italy, *n* = 117 [8%]; Spain, *n* = 158 [11%]; UK, *n* = 155 [11%]; USA, *n* = 506 [36%]). A total of 483 patients (34%) met the criteria for insufficient response; the remaining 930 patients (66%) were TRs (Fig. 1). A total of 615 physicians participated in the study (France, *n* = 98 [16%]; Germany, *n* = 91 [15%]; Italy, *n* = 92 [15%]; Spain, *n* = 92 [15%]; UK, *n* = 90 [15%]; USA, *n* = 152 [25%]. Of these, 359 were PCPs (France, *n* = 54 [15%]; Germany, *n* = 51 [14%]; Italy, *n* = 51 [14%]; Spain, *n* = 52 [14%]; UK, *n* = 50 [14%]; USA, *n* = 101 [28%] and 256 were neurologists or headache specialists (France, *n* = 44 [17%]; Germany, *n* = 40 [16%]; Italy, *n* = 41 [16%]; Spain, n = 40 [16%]; UK, n = 40 [16%]; USA, n = 51 [20%]. The majority of patients were seen by a PCP at the time of the current consultation (*n* = 1001; 71%); the remaining 412 patients (29%) had consulted a neurologist or headache specialist.

Patient characteristics are summarized in Table 1. TIRs were significantly more likely than TRs to be older, female, and to have had a longer mean time since diagnosis (6.6 versus 5.3 years, respectively; *p* = 0.026). Physician-assessed current cardiovascular risk was significantly higher in TIRs. TIRs were also more likely to have experienced (previously or currently) cardiovascular disease. The most common cardiovascular conditions currently and previously experienced by patients are summarized in Supplementary Table 1. TRs had a mean of 1.0 comorbid condition, whereas TIRs had a mean of 1.6 comorbidities. Anxiety, stress, sleeping disorder, and depression were significantly more prevalent in TIRs versus TRs (all *p* < 0.01).

**Table 1:** Patient demographic and clinical characteristics (physician reported)

Characteristic	TIRs (*n* = 483)	TRs (*n* = 930)	All (*n* = 1413)	*p*-value^a^
Mean age, years (±SD)	42.6 (±13.1)	40.5 (±12.8)	41.2 (±13.0)	0.003
Female, n (%)	368 (76.2)	649 (69.8)	1017 (72.0)	0.011
Country				0.004
France	85 (17.6)	110 (11.8)	195 (13.8)	
Germany	93 (19.3)	189 (20.3)	282 (20.0)	
Italy	46 (9.5)	71 (7.6)	117 (8.3)	
Spain	61 (12.6)	97 (10.4)	158 (11.2)	
UK	52 (10.8)	103 (11.1)	155 (11.0)	
USA	146 (30.2)	360 (38.7)	506 (35.8)	
Treating physician speciality^b^				0.061
PCP/GP	327 (67.7)	674 (72.5)	1001 (70.8)	
Neurologist	156 (32.3)	256 (27.5)	412 (29.2)	
Concomitant conditions, n (%)				
Anxiety	145 (30.0)	167 (18.0)	312 (22.1)	< 0.001
Stress	83 (17.2)	113 (12.2)	196 (13.9)	0.009
Depression	95 (19.7)	85 (9.1)	180 (12.7)	< 0.001
Sleeping disorder, n (%)	40 (8.3)	36 (3.9)	76 (5.4)	< 0.001
Current cardiovascular risk, n (%)^c^				< 0.001
Mild	364 (80.0)	757 (87.3)	1121 (84.8)	
Moderate	81 (17.8)	103 (11.9)	184 (13.9)	
Severe	10 (2.2)	7 (0.8)	17 (1.3)	
Cardiovascular risk at start of acute migraine medication, n (%)^c^				0.003
Mild	355 (80.7)	727 (87.5)	1082 (85.1)	
Moderate	71 (16.1)	92 (11.1)	163 (12.8)	
Severe	14 (3.2)	12 (1.4)	26 (2.0)	
Current cardiovascular condition, n (%)	109 (22.6)	142 (15.3)	251 (17.8)	< 0.001
Past cardiovascular condition, n (%)	106 (21.9)	125 (13.4)	231 (16.3)	< 0.001
Employment characteristics				
Currently employed, n (%)	318 (68.7)	633 (69.6)	951 (69.3)	0.739
Mean No. of hours worked/per week (SD)	36.6 (9.5)	36.6 (9.6)	36.6 (9.6)	0.951
Mean No. of work days missed because of doctors’ appointments, (±SD)^d^	1.09 (±1.88)	0.60 (±2.28)	0.76 (±2.17)	< 0.001
Mean No. of work days missed because of migraine, (±SD)^d^	2.12 (±3.81)	0.89 (±2.43)	1.29 (±3.01)	< 0.001

^a^P-values from test of two group difference: t-test for continuous variables and chi-square for categorical variables. ^b^Physician currently managing the patient. ^c^Subjective opinion of the physician.^d^During the past 3 months

SD = standard deviation; TIR = triptan insufficient responder; TR = triptan responder

### Migraine characteristics

Patients in the total population had a physician-reported mean of 5.3 monthly headache days over the last 3 months (Table 2). TIRs experienced more monthly headache days than TRs: physicians reported a mean of 7.0 versus 4.4, respectively, whereas patients reported 7.3 versus 4.3, respectively (both *p* ≤ 0.001). TIRs were more likely to have chronic migraine (≥15 HDMs) than TRs (11.6% of TIRs versus 3.2% of TRs; *p* < 0.001). Symptoms associated with migraine, including sensitivity to light, sound, and smell, nausea, vomiting, and bilateral pain, were significantly more common in TIRs than TRs (Table 2).

**Table 2:** Migraine characteristics

Characteristic	TIRs (*n* = 483)	TRs (*n* = 930)	All (*n* = 1413)	*p*-value^a^
Diagnosis, n (%)^b,c^				
Migraine without aura	254 (52.6)	509 (54.7)	763 (54.0)	0.443
Migraine with aura	201 (41.6)	363 (39.0)	564 (39.9)	0.347
Menstrual/menstrual-related migraine	54 (11.2)	84 (9.0)	138 (9.8)	0.197
Other	6 (1.2)	4 (0.4)	10 (0.7)	0.100
Migraine diagnosis based on ICHD criteria, n (%)				0.145
Yes	253 (52.4)	436 (46.9)	689 (48.8)	
No	125 (25.9)	271 (29.1)	396 (28.0)	
Don’t know	105 (21.7)	223 (24.0)	328 (23.2)	
Migraine severity^d^ over past 3 months, mean (SD)				
Patient reported	6.6 (1.8)	5.5 (2.1)	5.9 (2.0)	< 0.001
Physician reported	6.2 (1.9)	5.2 (2.0)	5.6 (2.0)	< 0.001
Headache days per month over past 3 months, mean (SD)				
Patient reported	7.3 (±5.8)	4.3 (±3.8)	5.3 (±4.8)	< 0.001
Physician reported	7.0 (± 5.9)	4.4 (±4.1)	5.3 (±4.9)	< 0.001
Chronic migraine (physician reported ≥15 headache days per month, over the past 3 months), n (%)^c^	56 (11.6)	30 (3.2)	86 (6.1)	< 0.001
Timing of migraine, n (%)^e^				
Morning (7 am–midday)	89 (18.8)	207 (22.6)	296 (21.3)	0.105
Afternoon (midday–5 pm)	71 (15.0)	145 (15.8)	216 (15.5)	0.696
Evening (5 pm–10 pm)	81 (17.1)	130 (14.2)	211 (15.2)	0.147
During the night (10 pm-7 am)	40 (8.5)	46 (5.0)	86 (6.2)	0.012
Occur at different times	266 (56.2)	489 (53.3)	755 (54.3)	0.302
Do not know	15 (3.2)	20 (2.2)	35 (2.5)	0.264
Current symptoms, n (%)^e^				
Pulsating/throbbing pain	369 (77.2)	683 (73.8)	1052 (75.0)	0.169
Sensitivity to light	347 (72.6)	620 (67.0)	967 (68.9)	0.033
Pain on one side of the head	318 (66.5)	648 (70.1)	966 (68.9)	0.176
Nausea	349 (73.0)	606 (65.5)	955 (68.1)	0.004
Sensitivity to sound	297 (62.1)	414 (44.8)	711 (50.7)	< 0.001
Pain worsened by activity/movement	242 (50.6)	350 (37.8)	592 (42.2)	< 0.001
Pain on both sides of the head	231 (48.3)	339 (36.6)	570 (40.6)	< 0.001
Vomiting	176 (36.8)	250 (27.0)	426 (30.4)	< 0.001
Sensitivity to smell	180 (37.7)	208 (22.5)	388 (27.7)	< 0.001
Visual aura/sight disturbance	166 (34.7)	191 (20.6)	357 (25.4)	< 0.001
Light-headedness/dizziness	144 (30.1)	151 (16.3)	295 (21.0)	< 0.001
Muscle weakness/fatigue	143 (29.9)	151 (16.3)	294 (21.0)	< 0.001
Pins needles/numbness	93 (19.5)	108 (11.7)	201 (14.3)	< 0.001
Kinesiophobia	76 (15.9)	73 (7.9)	149 (10.6)	< 0.001
Speech disturbance/ problems	49 (10.3)	36 (3.9)	85 (6.1)	< 0.001
Allodynia	37 (7.7)	41 (4.4)	78 (5.6)	0.010

^a^*P*-values from test of two group difference: t-test for continuous variables and chi-square for categorical variables. ^b^Physicians could attribute more than one diagnosis to each patient if applicable. ^c^Physician reported. ^d^Reported severity using scale from 1 (very mild) to 10 (very severe). ^e^Patient reported

SD = standard deviation; TIR = triptan insufficient responder; TR = triptan responder

### Treatment characteristics

All patients in this analysis were currently receiving any prescribed triptan as their only acute medication for their migraine. As shown in Table 3, the most common medications were sumatriptan, rizatriptan, and zolmitriptan; TIRs were more likely to have tried a higher number of triptans than TRs. Patients who took sumatriptan were more likely to be TRs than TIRs, and those who took naratriptan and frovatriptan were more likely to be TIRs. TRs were more likely to take their medication at the first sign of an attack (i.e. premonitory phase) compared with TIRs (Table 3).

**Table 3:** Migraine treatment characteristics

Treatment, n (%)	TIRs (*n* = 483)	TRs (*n* = 930)	All (*n* = 1413)	*p*-value^a^
Triptan prescribed^b,c^				
Sumatriptan	194 (40.2)	435 (46.8)	629 (44.5)	0.018
Sumatriptan + naproxen	24 (5.0)	55 (5.9)	79 (5.6)	0.463
Rizatriptan	85 (17.6)	157 (16.9)	242 (17.1)	0.734
Zolmitriptan	82 (17.0)	147 (15.8)	229 (16.2)	0.571
Almotriptan	19 (3.9)	57 (6.1)	76 (5.4)	0.083
Eletriptan	33 (6.8)	42 (4.5)	75 (5.3)	0.065
Naratriptan	34 (7.0)	27 (2.9)	61 (4.3)	< 0.001
Frovatriptan	18 (3.7)	11 (1.2)	29 (2.1)	0.001
No. of unique triptans ever used, n (%)^b^				< 0.001
1	389 (80.5)	824 (88.6)	1213 (85.8)	
2	80 (16.6)	98 (10.5)	178 (12.6)	
3+	14 (2.9)	8 (0.9)	22 (1.6)	
Timing of acute medication use^d^				< 0.001
At the first sign of a migraine attack (premonitory phase; before pain starts)	276 (58.2)	641 (70.5)	917 (66.3)	
When the pain starts	162 (34.2)	238 (26.2)	400 (28.9)	
After the pain has started and severity is known	36 (7.6)	30 (3.3)	66 (4.8)	
Current use of OTC migraine medication^d^	100 (21.8)	140 (15.6)	240 (17.7)	0.005
Mean amount spent in last month on OTC products, $ (SD)^e^	12.78 (17.55)	10.54 (9.34)	11.42 (13.21)	0.294
Reason for OTC use^d^				
Prescribed medication not effective enough	47 (50.5)	37 (29.1)	84 (38.2)	0.001
My doctor recommended it	22 (23.7)	47 (37.0)	69 (31.4)	0.035
I run out of my prescribed medication	20 (21.5)	14 (11.0)	34 (15.5)	0.034
Prescribed medication is too expensive	9 (9.7)	11 (8.7)	20 (9.1)	0.796
Current use of preventive medication^b^	262 (54.2)	390 (41.9)	652 (46.1)	< 0.001
Anticonvulsants	102 (21.1)	106 (11.4)	208 (14.7)	< 0.001
Antidepressants/anxiolytics/benzodiazepines	48 (9.9)	70 (7.5)	118 (8.4)	0.120
Antimigraine	8 (1.7)	8 (0.9)	16 (1.1)	0.180
Beta blockers	89 (18.4)	159 (17.1)	248 (17.6)	0.533
Calcium antagonists	14 (2.9)	36 (3.9)	50 (3.5)	0.348
NSAIDS, including combinations	4 (0.8)	7 (0.8)	11 (0.8)	1.000
Others	16 (3.3)	19 (2.0)	35 (2.5)	0.145
History of opioid misuse, n (%)^b^	19 (3.9)	20 (2.2)	39 (2.8)	0.019

^a^*P*-values from test of two group difference: t-test for continuous variables and chi-square for categorical variables. ^b^Physician reported. ^c^Patients could be receiving more than one triptan, but no other acute medication type. ^d^Patient reported. ^e^Average currency exchange rate at year 2017 was used to convert to US dollar amounts. Website referred to: https://www.irs.gov/individuals/international-taxpayers/yearly-average-currency-exchange rates

*NSAID* nonsteroidal anti-inflammatory drug; *OTC* over-the-counter; *TIR* triptan insufficient responder; *TR* triptan responder

Preventive medication was currently prescribed to 46% of patients overall, with significantly more TIRs than TRs currently receiving preventive agents; topiramate, propranolol, and metoprolol were the most commonly prescribed preventive agents (preventive monoclonal antibodies were not yet marketed at the time of this survey).

OTC medication use was significantly greater in TIRs than TRs (Table 3). Significantly more TIRs than TRs reported insufficient efficacy of prescribed medication and running out of prescribed medication as reasons for OTC medication use, whereas significantly more TRs than TIRs cited physician recommendation as the reason for using OTC medication.

**Fig. 2:**
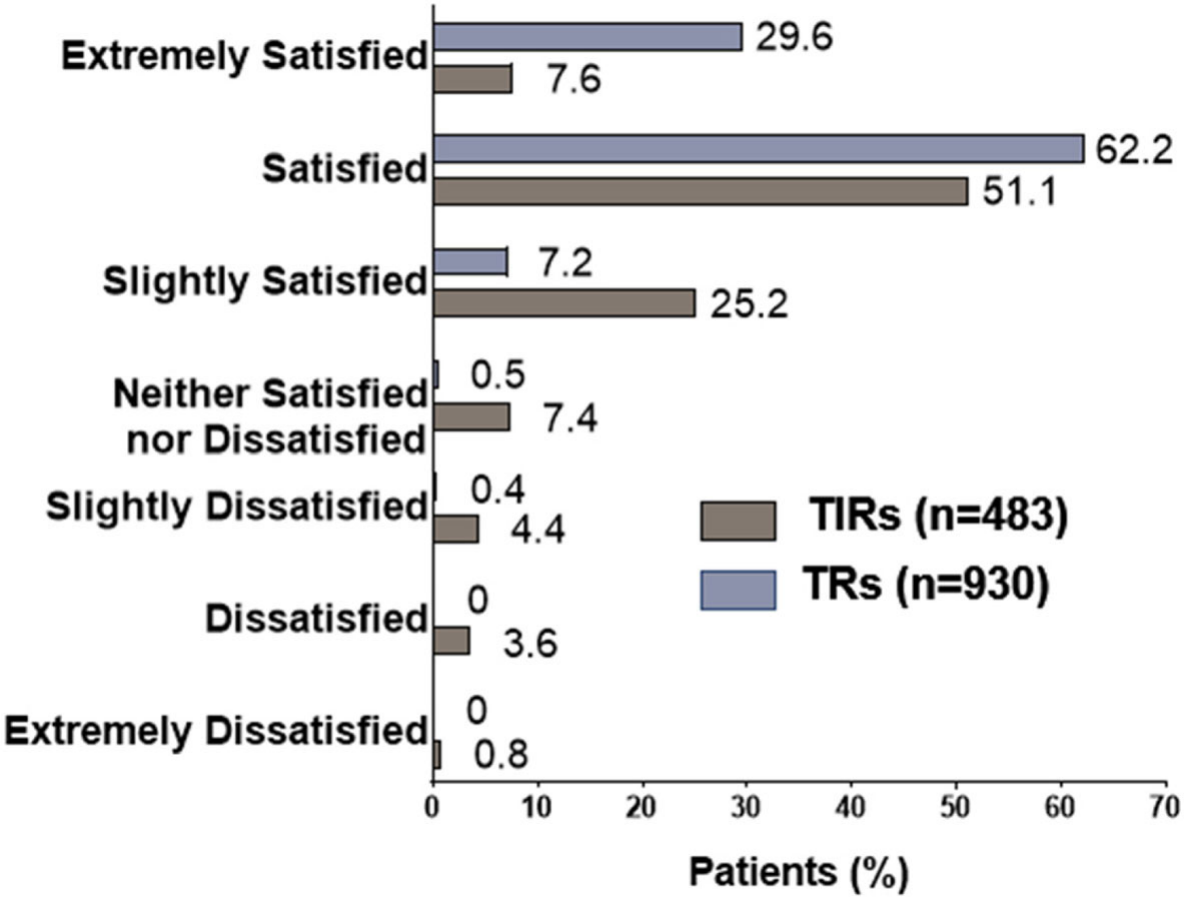
Patient-reported satisfaction with acute medication. Note: *p* < 0.001 overall for TIRs versus TRs using Fisher’s exact test. TIR = triptan insufficient responder; TR = triptan responder

Patient-reported satisfaction with treatment was significantly greater in TRs versus TIRs (*p* < 0.001; Fig. 2). The most common reasons for dissatisfaction in TIRs, as reported by patients, were lack of efficacy and medication not working as well as it used to; the most common reason for dissatisfaction among TRs was medication not working as well as it used to (Table 4).

**Table 4:** Satisfaction with medication for acute relief of migraine symptoms (patient reported)

Treatment, n (%)	TIRs (n = 483)	TRs (n = 930)	All (n = 1413)	*p*-value^a^
Would you like to continue taking your medication?^b^				< 0.001
Definitely yes	169 (37.0)	604 (69.1)	773 (58.1)	
Probably yes	209 (45.7)	243 (27.8)	452 (34.0)	
Do not know	59 (12.9)	24 (2.7)	83 (6.2)	
Probably not	16 (3.5)	3 (0.3)	19 (1.4)	
Definitely not	4 (0.9)	0	4 (0.3)	
Reasons for dissatisfaction^b,c^				
I feel that my migraine medication doesn’t work well	79 (58.5)	12 (31.6)	91 (52.6)	0.003
My migraine medication does not work as well as it used to	46 (34.1)	14 (36.8)	60 (34.7)	0.751
The severity of the side effects I experience	23 (17.0)	2 (5.3)	25 (14.5)	0.068
The number of side effects I experience	14 (10.4)	2 (5.3)	16 (9.2)	0.528
I am not happy with the way I have to take my migraine medication	10 (7.4)	5 (13.2)	15 (8.7)	0.325
Cost	1 (0.7)	0	1 (0.6)	1.00
Other reasons	5 (1.2)	6 (0.7)	11 (0.8)	0.344

^a^*P*-values from test of two group difference: t-test for continuous variables and chi-square for categorical variables. ^b^Patient reported. ^c^In patients who were neither satisfied nor extremely satisfied with acute medication. Patients could tick all responses that applied

*TIR* triptan insufficient responder; *TR* triptan responder

### HRQoL

TIRs experienced a significantly greater HRQoL burden than TRs across a range of outcome measures (Supplementary Table 2) and after controlling for age, sex, migraine frequency, comorbidities, duration of illness, preventive medication use, and presence of aura (Supplementary Table 3).

**Fig. 3:**
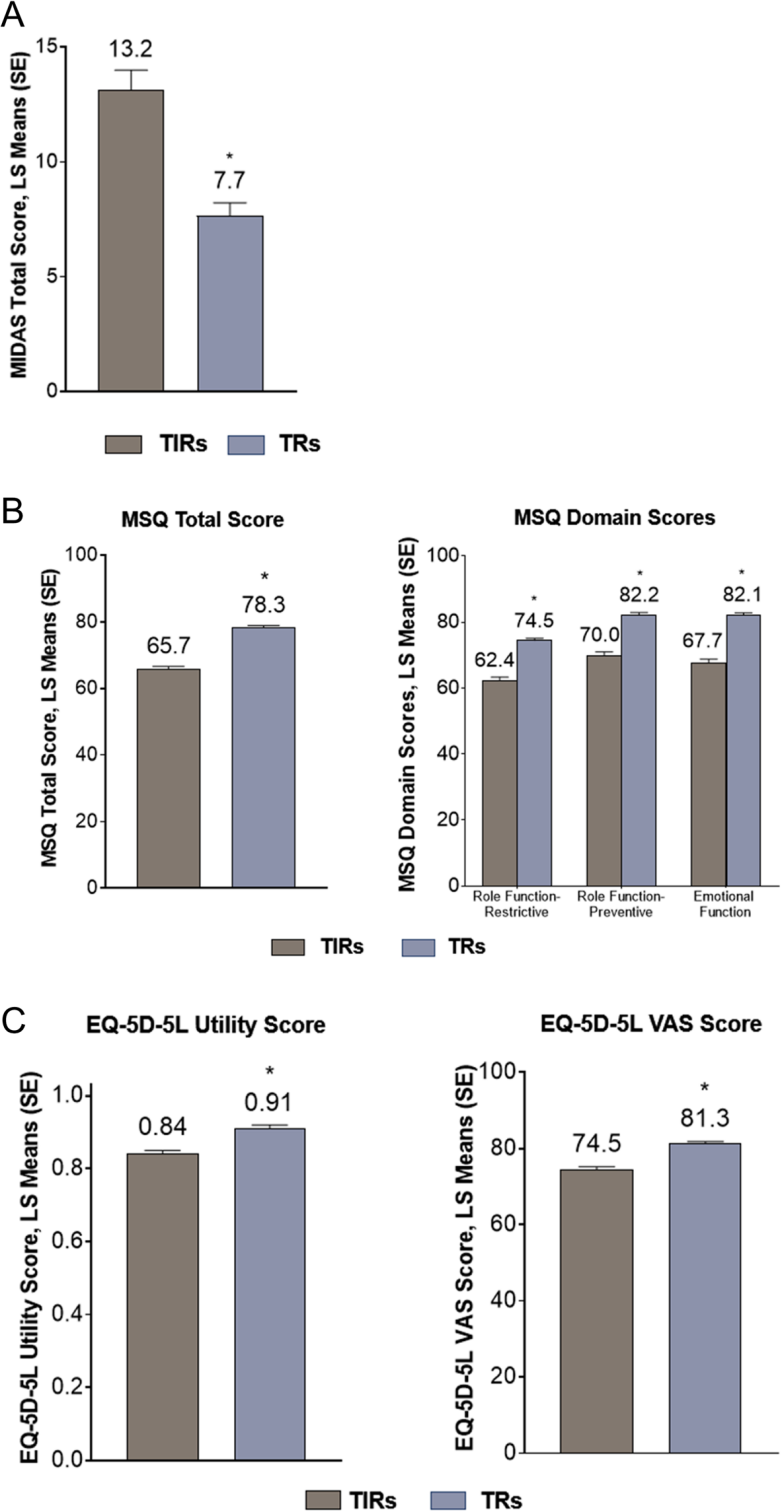
Patient-reported outcomes in patients with migraine receiving treatment for acute migraine: **a** MIDAS; **b** MSQ; and **c** EQ-5D-5L scores according to response to triptan medication. Data were controlled for age, sex, migraine headache day frequency, comorbidities, duration of illness, preventive medication use, and presence of aura. *p < 0.001 for TIRs versus TRs using multivariable general linear model controlling for age, sex, migraine headache day frequency, comorbidities, duration of illness, preventive medication use, and migraine with or without aura. Note: Higher MIDAS score indicates greater disability; lower MSQ score indicates lower HRQoL; lower EQ-5D-5L score indicates lower HRQoL. EQ-5D-5L = EuroQol 5-Dimensions 5-Levels questionnaire; HRQoL = health-related quality of life; LS = least squares; MIDAS = Migraine Disability Assessment; MSQ = Migraine-Specific Quality of Life Questionnaire; SE = standard error; TIR = triptan insufficient responder; TR = triptan responder, VAS = visual analog scale

A higher level of migraine-related disability was reported by TIRs based on MIDAS responses: TIRs had adjusted mean score of 13.2 (SE 0.8) compared with 7.7 (SE 0.6) for TRs (*p* < 0.001). (Fig. 3A). The MSQ indicated a statistically significantly greater impact in TIRs across all MSQ domains compared with TRs after adjustment (Fig. 3B). EQ-5D utility and VAS scores were also significantly lower in TIRs versus TRs indicating worse HRQoL (*p* < 0.001; Fig. 3C).

**Fig. 4:**
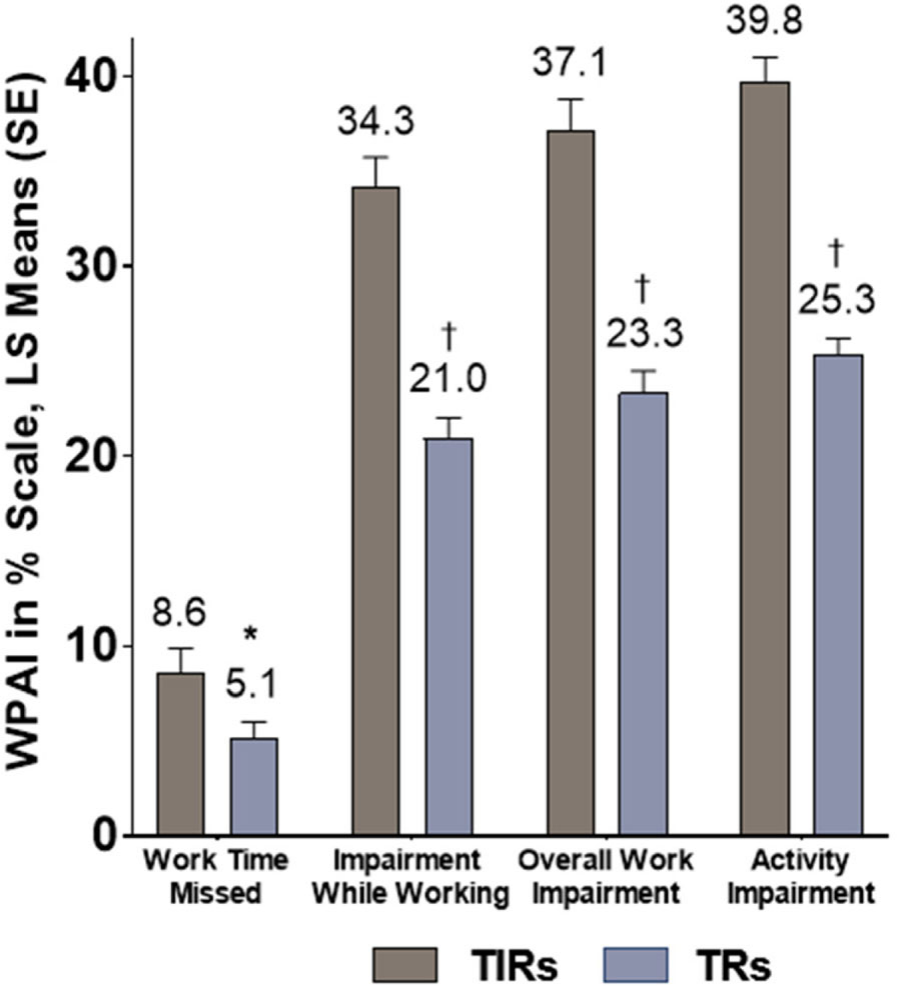
Patient-reported work productivity and activity impairment according to patient response to triptan medication. *p < 0.05; ^**†**^*p* < 0.001 for TIRs versus TRs using multivariable general linear model controlling for age, sex, migraine headache day frequency, comorbidities, duration of illness, preventive medication use, and migraine with or without aura. Note: Higher WPAI score indicates greater impairment. LS = least squares; WPAI=Work Productivity and Activity Impairment

Insufficient response to triptan medication had a significant impact on work productivity and activity. Absenteeism, presenteeism, work impairment, and overall activity impairment, as measured using the WPAI, were significantly higher among TIRs than TRs (all *p* < 0.05; Fig. 4). TIRs reported missing more workdays due to migraines and/or migraine-related doctors’ appointments than TRs (2.1 and 1.1 days vs. 0.9 and 0.6 days, respectively, *p* < 0.001).

### Characteristics associated with insufficient response

Backward logistic regression revealed the factors associated with TIR (Table 5).

**Table 5:** Logistic regression modeling triptan insufficient response with backward selection results. P-values in bold indicate statistically significant factors (*N* = 1033)

	Odds ratio estimates			
Factor	Effect	Point estimate	95% Wald confidence intervals	*p*-value
Age^a^		0.999	0.988, 1.011	0.930
Sex^a^	Female vs Male	0.920	0.663, 1.277	0.612
Currently taking OTC medications for migraines^a^	Yes vs No	**1.465**	**1.000, 2.147**	**0.050**
When do you usually take your prescribed acute medication?^a^	After pain has started and I have an idea of how severe it is vs At first sign of a migraine (before pain starts)	**2.433**	**1.258, 4.703**	**0.008**
	When pain starts vs At first sign of a migraine (before pain starts)	**1.726**	**1.252, 2.380**	**0.001**
On average, per month over the last 3 months: No. of migraine-related headache days^b^		0.926	0.856, 1.002	0.057
MSQ domain: Preventive^a^		**0.980**	**0.968, 0.992**	**0.001**
MSQ domain: Emotional^a^		**0.981**	**0.970, 0.992**	**0.001**
EQ-5D utility scores^a^		**0.106**	**0.032, 0.355**	**< 0.001**
Total headache days (including migraine, tension, rebound, etc.) per month in last 3 months^b^		**1.095**	**1.023, 1.174**	**0.010**
How severe have the patient’s migraine attacks been over the last 3 months?^b^		**1.025**	**1.003, 1.048**	**0.027**

^a^Patient reported. ^b^Physician reported

The results in the table are from a backward logistic regression with a significance level of 0.1 for a variable to stay in the model. A total of 380 patients (380/1413; 26.8%) were excluded from the analyses due to missingness of variables

EQ-5D = EuroQol 5-Dimensions questionnaire; MSQ = Migraine-Specific Quality of Life Questionnaire; OTC = over-the-counter

When compared with patients not taking OTC medications for their migraine, patients taking OTC medications were more likely to be TIRs (OR 1.465; 95% CI 1.000, 2.147; *p* = 0.050). Compared with patients taking their prescribed acute medication at first the sign of a migraine attack (i.e. premonitory phase), patients taking medication after the pain has started were more likely to be TIRs (OR 2.433; 95% CI 1.258, 4.703; *p* = 0.008). Similarly, patients taking medication when the pain starts were more likely to be TIRs than those taking their prescribed acute medication at the first sign of a migraine attack (i.e. premonitory phase) (OR = 1.726; 95% CI 1.252, 2.380; *p* = 0.001). The likelihood of being a TIR was also associated with lower MSQ Preventive (OR = 0.980; 95% CI 0.968, 0.992; *p* = 0.001) and lower MSQ Emotional (OR = 0.981; 95% CI 0.970, 0.992; *p* = 0.001); per unit increment of MSQ score, the likelihood of being a TIR was reduced by 2% for MSQ Preventive and 1.9% for MSQ Emotional, and EQ-5D utility score (OR = 0.106; 95% CI 0.032, 0.355; *p* < 0.001); per unit increment of EQ-5D utility scores, the likelihood of being a TIR was reduced by 89%. Also positively associated with being a TIR were total headache days, on average, over the last 3 months (OR = 1.095; 95% CI 1.023, 1.174; *p* = 0.010), with a 9.5% increase of likelihood per unit increment of Total headache days, and more severe migraine attacks over the past 3 months (OR 1.025; 95% CI 1.003, 1.048; *p* = 0.027), with a 2.5% increase per unit increment of severity score.

## Discussion

The results of this real-world study of consulting patients with migraine currently treated with triptans show that those with an insufficient response to triptan medication have significantly worse HRQoL and higher work productivity burden than those who respond to triptans. This includes a higher level of disability according to MIDAS, a greater impact across all MSQ domains, lower EQ-5D-5L utility scores, and greater absenteeism and presenteeism.

TIRs, who comprised one-third of the population, had significantly more monthly headache days and greater migraine severity over the last 3 months than TRs. In their study of responders and nonresponders to sumatriptan, Visser and colleagues also reported more severe migraine symptoms, with nonresponders reporting more severe vomiting and photophobia, more often needing to sleep or rest, and more frequently experiencing initial worsening of the headache after oral sumatriptan administration than responders [28]. Underlying disease severity, with some patients being difficult to treat, may be a reason for the poor response to triptans among TIRs.

Patients consider complete relief of headache pain (i.e. pain freedom), no recurrence, and rapid onset of action to be important attributes of migraine medication [29]; patients also want to be able to function again quickly [30]. Patients with chronic migraine present a greater therapeutic challenge than those with episodic migraine, particularly when chronic migraine has evolved from ineffectively treated episodic migraine [18]. Patients with chronic migraine have a higher rate of comorbidities, including anxiety, depression, and insomnia [31], which may impact on adherence to treatment. Patients with chronic migraine often find it difficult to know when to take their acute migraine medication, potentially impacting on the efficacy of these treatments [32]. The potential contribution of these factors is difficult to assess in a cross-sectional study, such as the present study, because no causal inferences can be drawn from the data obtained.

The measure used to determine effectiveness of triptan medication in this study was freedom from pain 2 h after taking medication, in at least four out of five attacks. This is a robust clinical endpoint, addressing the attributes considered important by patients when determining response to treatment [33]. Another important consideration is the consistency of pain relief, although measures used to define this are varied: Viana and colleagues defined this as a positive outcome in at least two out of three treated attacks [12], whereas Ho and colleagues required pain relief in three out of four attacks [34]. We previously used a definition of insufficient response to acute migraine treatment that was anchored on pain freedom at 2 h, while also reflecting consistency of response at a threshold above the most conservative estimates in the literature, i.e. four out of five attacks [35]. As freedom from pain at 2 h and consistency of response are important outcomes for patients, we believe that this definition of response to triptan medication was appropriate for the present study.

Differences in medication use were observed between TIRs and TRs. TIRs had received significantly more unique triptans than TRs, indicating a pattern of cycling through acute medications. This suggests that there may be little benefit in patients trying more than two triptans, consistent with recent guidance issued by the American Headache Society that patients who have failed two oral triptans are eligible for new acute therapies [36]. Preventive medication use was high overall in this population, with almost half of patients currently using preventive treatments. This is higher than the 12% of patients in the Migraine in America Symptoms and Treatment (MAST) study who reported using preventive medication [37], likely a consequence of our study including patients actively consulting their physician, in contrast to the less stringent inclusion criteria for the MAST study. Preventive medication use in the present study was statistically significantly higher among TIRs than TRs, which is not surprising given the greater disease severity and headache day frequency among the TIRs, and is in line with treatment guidelines that recommend preventive medication for patients not responding to, overusing, or experiencing frequent headache days despite, acute treatment [36]. Young and colleagues previously observed greater use of preventive medication in patients with chronic versus episodic migraine in their study of patients in community practice in the USA, most of whom had been offered triptan medication [38].

OTC medication use was significantly higher among TIRs than TRs. Our findings suggest that patients who do not respond adequately to triptans may be, to a greater extent than those with a good response, using OTC medication when their triptan fails to relieve their migraine, or using OTC medications in combination with their triptan. Patients may also use OTC medication if they do not have their triptan with them. Use of OTC medication, which has been shown to be common among patients with migraine [37, 39], can be concerning if inadequate control of symptoms or overuse results [37]. Overuse of OTC medication, including caffeine-containing products, and other acute medications such as barbiturates and opioids has been linked with migraine chronification [40].

Patient-reported satisfaction with treatment was generally good; however, patient-reported dissatisfaction was higher among TIRs than TRs. Lack of efficacy or diminished efficacy over time were the reasons most commonly given for dissatisfaction with treatment. Others have shown that fast and complete pain relief is associated with treatment satisfaction [41]. As reported by Cady and colleagues, predictors of sustained use of triptans include satisfaction with the medication, confidence that the triptan would resolve the headache, the reliability of the triptan to be effective on a regular basis, and fewer doses required to control the headache [42].

The impact of insufficient response to acute medication was reflected in statistically significantly lower HRQoL scores in the TIR population. These patients had greater migraine-related disability and worse migraine-specific quality of life, which was both statistically significant and clinically meaningful [43, 44]. Additionally, TIRs had lower EQ-5D scores than TRs. In their study of European patients with migraine who experienced ≥4 monthly headache days, Vo and colleagues reported an EQ-5D score of 0.68 for patients with migraine, somewhat lower than the scores reported in the present study (0.84 and 0.91 for TIRs and TRs, respectively) [45]. Unlike the present study, in which all patients were receiving treatment with triptans, 31–51% of patients in the study by Vo and colleagues were not receiving any prescription medication, which may have affected their HRQoL. TIRs in the present study also had more comorbidities than TRs, as reported by others [46]. There is likely a synergistic component, with lack of response and comorbidities affecting HRQoL scores [35]. Psychiatric comorbidities alone add to the burden experienced by patients with migraine [47], highlighting a need for effective treatment to reduce this aspect of patient burden.

Patients with an insufficient response to triptans in this study missed more workdays over the past 3 months because of doctors’ appointments and migraine attacks than those with a good response. TIRs also had reduced work productivity, as measured by the WPAI, missing 10% of work time over the past week compared with 5% in TRs. Increasing migraine severity, as measured by monthly migraine headache days, was shown to be associated with increased acute medication use and reduced work productivity (absenteeism and presenteeism) in an analysis of three randomized controlled trials [48]. Notably, the relationship was nonlinear in that study: a patient with eight monthly migraine headache days was predicted to have 0.95 absenteeism days and 2.34 presenteeism days per month, whereas a patient with 18 monthly migraine headache days was predicted to have 1.86 absenteeism days and 5.18 presenteeism days per month. Vo and colleagues reported significantly increased absenteeism and presenteeism in people with migraine compared with healthy controls [45]. These findings suggest high societal and economic costs of migraine in patients who respond inadequately to their acute triptan medication. Based on data from the American Migraine Prevalence and Prevention Study, Serrano and colleagues estimated that lost productive time in patients with migraine cost between $43 and $137 per week for women aged 25–64 years, and between $54 and $276 per week for men [49]; costs were higher in those with chronic versus episodic migraine at every age and in both sexes. These data suggest that addressing the issue of nonresponse to triptans has the potential to improve work productivity and reduce the economic impact of migraine in this challenging patient population.

Logistic regression analysis identified several factors as being statistically significantly associated with an insufficient response to triptan medication in this study. These were related to: medication use, namely the timing of triptan administration (taking the triptan when/after pain has started versus at first sign of a migraine attack, i.e. the premonitory phase) and use of OTC medication; HRQoL scores (MSQ Preventive and Emotional domains and EQ-5D total scores); and headache frequency (higher number of monthly headache days, on average, over the last 3 months) and severity (more severe migraine attacks over the last 3 months). Among these, only timing of acute medication and MIDAS Total Score were identified in our previous analysis of predictors of insufficient response to acute migraine medication among patients in the USA [34]. That study included patients receiving any acute medication for their migraine, whereas the present study included those receiving triptan medication only for the acute treatment of their migraine, allowing us to focus on response to triptans in the most appropriate population. Others have identified a number of sociodemographic, disease, and clinical factors associated with failure to achieve freedom from pain at 2 h after administration of acute migraine medication, including body mass index, female sex, allodynia, and depression [46]; consistency of response over a number of attacks was not considered in that analysis. The association of additional migraine features and response to triptan medication has implications for clinical trial design, as incorporation of “most bothersome symptom” is becoming increasingly common in migraine clinical studies [50]. FDA guidance for the development of drugs for acute treatment of migraine now includes the recommendation that studies should demonstrate an effect on both pain and the patient’s most bothersome symptom, recognizing the importance of treatment effect on symptoms of primary importance to patients [51].

Some limitations of this study should be considered. Physicians were recruited based on the volume of patients with migraine routinely seen under their care and were therefore experienced in the management of patients with migraine. Physicians were asked to provide data for a consecutive series of patients to avoid selection bias, but no formal patient selection verification procedures were in place. Identification of the target patient group was based on the judgement of the respondent physician and not a formalized diagnostic checklist; this is, however, representative of physician’s real-world classification of the patient. Patients had a diagnosis of migraine, and were actively seeking medical care; consequently, they may not be representative of the general migraine population. The study was exploratory and aimed to identify association between response to treatment and HRQoL and work productivity. As this was a cross-sectional study, no causal inferences can be made regarding the relationship between insufficient response to medication and deficits in HRQoL and work productivity. It is unknown if any of the characteristics identified are the cause of the insufficient response to triptan medication or if the insufficient response to triptans resulted in characteristics such as increased disability and reduced work productivity. Further longitudinal research would help elucidate interactions between these factors.

The present study also has strengths: these include a good sample size and inclusion of patients who are more representative of the general migraine population than those typically entered into randomized controlled trials, which may exclude older patients, those with comorbidities, and those with severe disease. For measures reported by both the patient and the physician, including migraine characteristics, results were generally similar, adding to the strength of the findings. An additional strength of the study was the large number of patient and disease characteristics collected and the range of outcomes assessed in the real-world clinical practice setting.

## Conclusion

This study has demonstrated that HRQoL and work productivity are significantly worse in patients with migraine who respond insufficiently to triptans than in those classified as TRs. Furthermore, logistic regression analysis identified an association between insufficient response to triptans and medication-related factors, including overuse of OTC medication and when patients take their triptan medication. Awareness of these factors may enable physicians to specifically ask patients about their migraine medication use and potentially identify those at risk of insufficient response to their triptan. Current migraine treatment guidelines, although contributing enormously to the effective management of patients with migraine, may need to be fine-tuned in order to guide a more holistic approach to the assessment and treatment of these patients. This may include acute and preventive treatments, in addition to nonpharmacological approaches such as lifestyle changes, and a change of treatment for patients who are not responding adequately to their current medication. Patient-reported outcomes tools, such as those described in this study and others such as the migraine Treatment Optimization Questionnaire (mTOQ) [52] may aid physicians in identifying patients in need of a treatment change in a timely manner. These findings highlight the need for more effective acute migraine treatment and a holistic approach to patient management to help alleviate the burden of migraine in patients who respond insufficiently to triptans.

## Supplementary information

**Additional file 1: Table 1.** Most frequently reported cardiovascular conditions experienced by patients according to response to triptan medication (physician-reported). **Table 2.** Summary of patient-reported outcomes in patients with migraine receiving treatment for acute migraine: unadjusted results. **Table 3.** Summary of patient-reported outcomes in patients with migraine receiving treatment for acute migraine; multivariate analysis controlling for age, gender, migraine headache day frequency, comorbidities, duration of illness, preventive medication use, with or without aura.

## Data Availability

The datasets generated and/or analysed during the current study are not publicly available as these are proprietary data. All of the conclusions drawn in the manuscript are based on data included in the publication and supporting literature has been provided.

## Abbreviations

5-HT: 5-Hydroxytryptamine
DSP: Disease Specific Programme
EphMRA: European Pharmaceutical Market Research Association
EQ-5D-5L: EuroQol 5-Dimensions 5-Levels questionnaire
HIPAA: Health Insurance Portability and Accountability Act
HITECH: Health Information Technology for Economic and Clinical Health
HRQoL: Health-related quality of life
MAST: Migraine in America Symptoms and Treatment
MIDAS: Migraine Disability Assessment questionnaire
MSQ: Migraine-Specific Quality of Life questionnaire
OR: Odds ratio
OTC: Over-the-counter
PCP: Primary care physician
PRF: Patient record form
SE: Standard error
TIR: Triptan insufficient responder
TR: Triptan responder
VAS: Visual analog scale
WPAI:Migraine: Work Productivity and Activity Impairment questionnaire:Migraine

## References

[1] Giffin NJ, Lipton RB, Silberstein SD, Olesen J, Goadsby PJ (2016). The migraine postdrome: an electronic diary study. Neurology.

[2] Burch RC, Loder S, Loder E, Smitherman TA (2015). The prevalence and burden of migraine and severe headache in the United States: updated statistics from government health surveillance studies. Headache.

[3] Stovner LJ, Andree C (2010). Prevalence of headache in Europe: a review for the Eurolight project. J Headache Pain.

[4] GBD 2017 Disease and Injury Incidence and Prevalence Collaborators (2018). Global, regional, and national incidence, prevalence, and years lived with disability for 354 diseases and injuries for 195 countries, 1990–2017: a systematic analysis for the Global Burden of Disease Study 2017. Lancet.

[5] Blumenfeld AM, Varon SF, Wilcox TK, Buse DC, Kawata AK, Manack A (2011). Disability, HRQoL and resource use among chronic and episodic migraineurs: results from the international burden of migraine study (IBMS). Cephalalgia..

[6] Ford JH, Jackson J, Milligan G, Cotton S, Ahl J, Aurora SK (2017). A real-world analysis of migraine: a cross-sectional study of disease burden and treatment patterns. Headache.

[7] Linde M, Dahlöf C (2004). Attitudes and burden of disease among self-considered migraineurs — a nation-wide population-based survey in Sweden. Cephalalgia..

[8] Smitherman TA, Burch R, Sheikh H, Loder E (2013). The prevalence, impact, and treatment of migraine and severe headaches in the United States: a review of statistics from national surveillance studies. Headache.

[9] Messali AJ, Yang M, Gillard P, Tsai K, Tepper SJ, Bloudek LM (2014). Treatment persistence and switching in triptan users: a systematic literature review. Headache.

[10] Ferrari MD, Goadsby PJ, Roon KI, Lipton RB (2002). Triptans (serotonin, 5-HT1B/1D agonists) in migraine: detailed results and methods of a meta-analysis of 53 trials. Cephalalgia.

[11] Mathew NT, Landy S, Stark S, Tietjen GE, Derosier FJ, White J (2009). Fixed-dose sumatriptan and naproxen in poor responders to triptans with a short half-life. Headache.

[12] Viana M, Genazzani AA, Terrazzino S, Nappi G, Goadsby PJ (2013). Triptan nonresponders: do they exist and who are they?. Cephalalgia.

[13] Cameron C, Kelly S, Hsieh SC, Murphy M, Chen L, Kotb A (2015). Triptans in the acute treatment of migraine: a systematic review and network meta-analysis. Headache..

[14] Gallagher RM, Kunkel R (2003). Migraine medication attributes important for patient compliance: concerns about side effects may delay treatment. Headache.

[15] Maassen Van Den Brink A, Saxena PR (2004). Coronary vasoconstrictor potential of triptans: a review of in vitro pharmacologic data. Headache.

[16] Gilmore B, Michael M (2011). Treatment of acute migraine headache. Am Fam Physician.

[17] GlaxoSmithKline. Sumatriptan Prescribing Information. 2017. https://www.gsksource.com/pharma/content/dam/GlaxoSmithKline/US/en/Prescribing_Information/Imitrex_Tablets/pdf/IMITREX-TABLETS-PI-PIL.PDF. Accessed March 2020

[18] Lipton RB, Fanning KM, Serrano D, Reed ML, Cady R, Buse DC (2015). Ineffective acute treatment of episodic migraine is associated with new-onset chronic migraine. Neurology.

[19] Buse DC, Rupnow MF, Lipton RB (2009). Assessing and managing all aspects of migraine: migraine attacks, migraine-related functional impairment, common comorbidities, and quality of life. Mayo Clin Proc.

[20] Bigal ME, Lipton RB (2009). The epidemiology, burden, and comorbidities of migraine. Neurol Clin.

[21] Anderson P, Benford M, Harris N, Karavali M, Piercy J (2008). Real-world physician and patient behaviour across countries: disease-specific Programmes – a means to understand. Curr Med Res Opin.

[22] Babineaux SM, Curtis BH, Holbrook T, Milligan G, Piercy J (2016). Evidence for validity of a national physician and patient-reported, cross-sectional survey in China and UK: the disease specific Programme. BMJ Open.

[23] Jhingran P, Osterhaus JT, Miller DW, Lee JT, Kirchdoerfer L (1998). Development and validation of the migraine-specific quality of life questionnaire. Headache.

[24] Herdman M, Gudex C, Lloyd A, Janssen M, Kind P, Parkin D (2011). Development and preliminary testing of the new five-level version of EQ-5D (EQ-5D-5L). Qual Life Res.

[25] Stewart WF, Lipton RB, Kolodner K, Liberman J, Sawyer J (1999). Reliability of the migraine disability assessment score in a population-based sample of headache sufferers. Cephalalgia.

[26] Stewart WF, Lipton RB, Dowson AJ, Sawyer J (2001). Development and testing of the migraine disability assessment (MIDAS) questionnaire to assess headache-related disability. Neurology.

[27] Reilly MC, Zbrozek AS, Dukes EM (1993). The validity and reproducibility of a work productivity and activity impairment instrument. Pharmacoeconomics.

[28] Visser WH, de Vriend RH, Jaspers NH, Ferrari MD (1996). Sumatriptan-nonresponders: a survey in 366 migraine patients. Headache.

[29] Lipton RB, Hamelsky SW, Dayno JM (1999). Acute migraine therapy: do doctors understand what patients with migraine want from therapy?. Headache.

[30] Smelt AF, Louter MA, Kies DA, Blom JW, Terwindt GM, van der Heijden GJ (2014). What do patients consider to be the most important outcomes for effectiveness studies on migraine treatment? Results of a Delphi study. PLoS One.

[31] Buse DC, Reed ML, Fanning KM, Bostic R, Dodick DW, Schwedt TJ (2020). Comorbid and co-occurring conditions in migraine and associated risk of increasing headache pain intensity and headache frequency: results of the migraine in America symptoms and treatment (MAST) study. J Headache Pain.

[32] Weatherall MW (2015). The diagnosis and treatment of chronic migraine. Ther Adv Chronic Dis.

[33] Lipton RB, Hamelsky SW, Dayno JM (2002). What do patients with migraine want from acute migraine treatment?. Headache..

[34] Ho AP, Dahlof CG, Silberstein SD (2010). Randomized, controlled trial of telcagepant over four migraine attacks. Cephalalgia.

[35] Lombard L, Ford J, Ye W, Nichols R (2018). A real-world analysis of unmet needs in migraine for responders vs. non-responders to acute treatment headache. Headache.

[36] American Headache Society (2019). The American headache society position statement on integrating new migraine treatments into clinical practice. Headache.

[37] Lipton RB, Munjal S, Alam A, Buse DC, Fanning KM, Reed ML (2018). Migraine in America symptoms and treatment (MAST) study: baseline study methods, treatment patterns, and gender differences. Headache.

[38] Young NP, Philpot LM, Vierkant RA, Rosedahl JK, Upadhyaya SG, Harris A (2019). Episodic and chronic migraine in primary care. Headache.

[39] Zhang Y, Dennis JA, Leach MJ, Bishop FL, Cramer H, Chung VCH (2017). Complementary and alternative medicine use among us adults with headache or migraine: results from the 2012 National Health Interview Survey. Headache.

[40] Bigal ME, Lipton RB (2009). Overuse of acute migraine medications and migraine chronification. Curr Pain Headache Rep.

[41] Davies GM, Santanello N, Lipton R (2000). Determinants of patient satisfaction with migraine therapy. Cephalalgia.

[42] Cady RK, Maizels M, Reeves DL, Levinson DM, Evans JK (2009). Predictors of adherence to triptans: factors of sustained versus lapsed users. Headache.

[43] Lipton RB, Desai P, Sapra S, Buse DC, Fanning KM, Reed ML (2017). How much change in headache-related disability is clinically meaningful? Estimating meaningful within person change in midas using data from the AMPP study. Headache.

[44] Cole JC, Lin P, Rupnow MF (2009). Minimal important differences in the migraine-specific quality of life questionnaire (MSQ) version. Cephalalgia.

[45] Vo P, Fang J, Bilitou A, Laflamme AK, Gupta S (2018). Patients' perspective on the burden of migraine in Europe: a cross-sectional analysis of survey data in France, Germany, Italy, Spain, and the United Kingdom. J Headache Pain.

[46] Lipton RB, Munjal S, Buse DC, Fanning KM, Bennett A, Reed ML (2016). Predicting inadequate response to acute migraine medication: results from the American Migraine Prevalence and Prevention (AMPP) study. Headache..

[47] Dresler T, Caratozzolo S, Guldolf K, Huhn JI, Loiacono C, Niiberg-Pikksööt T, Puma M, Sforza G, Tobia A, Ornello R, Serafini G, European Headache Federation School of Advanced Studies (EHF-SAS) (2019). Understanding the nature of psychiatric comorbidity in migraine: a systematic review focused on interactions and treatment implications. J Headache Pain.

[48] Porter JK, Di Tanna GL, Lipton RB, Sapra S, Villa G (2018) Costs of acute headache medication use and productivity losses among patients with migraine: insights from three randomized controlled trials. Pharmacoecon Open. 10.1007/s41669-018-0105-0 10.1007/s41669-018-0105-0 PMC6710313 30377991

[49] Serrano D, Manack AN, Reed ML, Buse DC, Varon SF, Lipton RB (2013). Cost and predictors of lost productive time in chronic migraine and episodic migraine: results from the American Migraine Prevalence and Prevention (AMPP) study. Value Health.

[50] Dodick DW, Tepper SJ, Friedman DI, Gelfand AA, Kellerman DJ, Schmidt PC (2018). Use of Most bothersome symptom as a Coprimary endpoint in migraine clinical trials: a post-hoc analysis of the pivotal ZOTRIP randomized, controlled trial. Headache.

[51] U.S. Department of Health and Human Services Food and Drug Administration Center for Drug Evaluation and Research (CDER). Migraine: Developing Drugs for Acute Treatment Guidance for Industry. Available at https://www.fda.gov/media/89829/download. Accessed March 2020

[52] Serrano D, Buse DC, Manack Adams A, Reed ML, Lipton RB (2015). Acute treatment optimization in episodic and chronic migraine: results of the American Migraine Prevalence and Prevention (AMPP) study. Headache.

