# Supplementary material for: A global real-world assessment of the impact on health-related quality of life and work productivity of migraine in patients with insufficient versus good response to triptan medication
Source: J Headache Pain. 2020 Apr 29;21(1):41. doi: 10.1186/s10194-020-01110-9 (PMC7189443; doi:10.1186/s10194-020-01110-9)
Supplement: Supplementary file 1 — Additional file 1: Table 1. Most frequently reported cardiovascular conditions experienced by patients according to response to triptan medication (physician-reported). Table 2. Summary of patient-reported outcomes in patients with migraine receiving treatment for acute migraine: unadjusted results. Table 3. Summary of patient-reported outcomes in patients with migraine receiving treatment for acute migraine; multivariate analysis controlling for age, gender, migraine headache day frequency, comorbidities, duration of illness, preventive medication use, with or without aura. [file 10194_2020_1110_MOESM1_ESM.docx]

### Supplementary Table 1 Most frequently reported cardiovascular conditions experienced by patients according to response to triptan medication (physician-reported)

| Characteristic | TIRs (n=483) | TRs (n=930) | All (n=1413) | *p*-value^a^ |
| --- | --- | --- | --- | --- |
| Cardiovascular condition (past), n (%)^b^ |  |  |  |  |
| Hypertension | 77 (15.9) | 91 (9.8) | 168 (11.9) | <0.001 |
| Hypotension | 8 (1.7) | 10 (1.1) | 18 (1.3) | 0.356 |
| Arrhythmia | 8 (1.7) | 7 (0.8) | 15 (1.1) | 0.116 |
| Ischemic heart disease | 6 (1.2) | 6 (0.6) | 12 (0.8) | 0.359 |
| Atrial fibrillation | 5 (1.0) | 7 (0.8) | 12 (0.8) | 0.557 |
| Deep vein thrombosis | 6 (1.2) | 4 (0.4) | 10 (0.7) | 0.100 |
| Reynaud’s disease | 6 (1.2) | 2 (0.2) | 8 (0.6) | 0.022 |
| Peripheral vascular disease | 4 (0.8) | 3 (0.3) | 7 (0.5) | 0.239 |
| Angina | 3 (0.6) | 2 (0.2) | 5 (0.4) | 0.345 |
| Cardiovascular condition (current), n (%)^b^ |  |  |  |  |
| Hypertension^c^ | 88 (18.2) | 105 (11.3) | 193 (13.7) | <0.001 |
| Hypotension | 7 (1.4) | 11 (1.2) | 18 (1.3) | 0.672 |
| Arrhythmia | 7 (1.4) | 7 (0.8) | 14 (1.0) | 0.258 |
| Ischemic heart disease | 7 (1.4) | 7 (0.8) | 14 (1.0) | 0.258 |
| Atrial fibrillation | 4 (0.8) | 7 (0.8) | 11 (0.8) | 1.000 |
| Reynaud’s disease | 5 (1.0) | 3 (0.3) | 8 (0.6) | 0.131 |
| Peripheral vascular disease | 3 (0.6) | 5 (0.5) | 8 (0.6) | 1.000 |
| Angina | 4 (0.8) | 2 (0.2) | 6 (0.4) | 0.189 |
| Deep vein thrombosis | 3 (0.6) | 2 (0.2) | 5 (0.4) | 0.345 |

^a^P-values from test of two group difference: t-test for continuous variables and chi-square for categorical variables. ^b^Subjective opinion of the physician. ^c^Hypertension was ‘somewhat controlled’ or ‘poorly controlled’ in 25 patients in the TIR group, 21 patients in the TR group, and 46 patients in the overall population.

TIR=triptan insufficient responder; TR=triptan responder

### Supplementary Table 2 Summary of patient-reported outcomes in patients with migraine receiving treatment for acute migraine: unadjusted results

| PROs, mean ± SD | TIRs (n=483) | TRs (n=930) | All (n=1413) | *p*-value^a^ |
| --- | --- | --- | --- | --- |
| MIDAS score | 15.85 ± 19.26 | 7.47 ± 12.2 | 10.16 ± 15.36 | <0.001 |
| MSQ score |  |  |  |  |
| Total | 62.17 ± 19.83 | 78.10 ± 16.83 | 72.67 ± 19.43 | <0.001 |
| Role function – Restrictive | 58.66 ± 20.20 | 74.18 ± 17.87 | 68.88 ± 20.09 | <0.001 |
| Role function – Preventive | 67.09 ± 20.65 | 82.07 ± 17.45 | 76.97 ± 19.90 | <0.001 |
| Emotional | 63.52 ± 24.46 | 81.96 ± 18.97 | 75.68 ± 22.74 | <0.001 |
| EQ-5D score^b^ |  |  |  |  |
| Utility | 0.82 ± 0.20 | 0.91 ± 0.11 | 0.881 ± 0.156 | <0.001 |
| VAS | 72.97 ± 17.39 | 82.64 ± 13.04 | 79.34 ± 15.36 | <0.001 |
| WPAI, % |  |  |  |  |
| Work time missed | 9.84 ± 21.15 | 4.843 ± 16.91 | 6.556 ± 18.62 | <0.001 |
| Impairment while working | 37.21 ± 26.3 | 20.99 ± 20.82 | 26.44 ± 24.05 | <0.001 |
| Overall work impairment | 40.66 ± 28.08 | 23.06 ± 22.87 | 29.02 ±26.11 | <0.001 |
| Activity impairment | 43.59 ± 26.16 | 25.2 ± 22.29 | 31.42 ± 25.21 | <0.001 |

^a^ P-values from test of two group difference: t-test for continuous variables and chi-square for categorical variables. ^b^Patients completed the EQ-5D 5L; scores were cross-walked to the 3L version [1, 2]. The number of patients with data varied by subgroup size.

EQ-5D=EuroQol 5-Dimensions questionnaire; MIDAS=Migraine Disability Assessment; MSQ=Migraine-Specific Quality of Life Questionnaire; SD=standard deviation; TIR=triptan insufficient responder; TR=triptan responder; VAS=visual analog scale; WPAI=Work Productivity and Activity Impairment.

### Supplementary Table 3 Summary of patient-reported outcomes in patients with migraine receiving treatment for acute migraine; multivariate analysis controlling for age, gender, migraine headache day frequency, comorbidities, duration of illness, preventive medication use, with or without aura

| PROs, LS mean ± SE (95% CI)^a^ | TIRs (n=483) | TRs (n=930) | *p*-value^b^ |
| --- | --- | --- | --- |
| MIDAS score | 13.15 ±0.84, (11.51,14.80) | 7.65 ±0.57, (6.53,8.78) | <0.001 |
| MIDAS category, n (%)^c^ |  |  | <0.001 |
| Little or no disability | 112 (29.4) | 500 (62.0) |  |
| Mild disability | 77 (20.2) | 119 (14.8) |  |
| Moderate disability | 108 (28.3) | 109 (13.5) |  |
| Severe disability | 84 (22.0) | 78 (9.7) |  |
| MSQ score |  |  |  |
| Total | 65.72 ±0.89, (63.98,67.45) | 78.31 ±0.63, (77.07,79.55) | <0.001 |
| Role function – Restrictive | 62.38 ±0.92, (60.57,64.19) | 74.46 ±0.66, (73.16,75.76) | <0.001 |
| Role function – Preventive | 70.01 ±0.94, (68.15,71.86) | 82.22 ±0.68, (80.89,83.54) | <0.001 |
| Emotional | 67.70 ±1.05, (65.65,69.75) | 82.10 ±0.75, (80.64,83.57) | <0.001 |
| EQ-5D score^d^ |  |  |  |
| Utility | 0.84 ±0.01, (0.83,0.86) | 0.91 ±0.01, (0.90,0.92) | <0.001 |
| VAS | 74.48 ±0.72, (73.07,75.88) | 81.29 ±0.51, (80.29,82.29) | <0.001 |
| WPAI, % |  |  |  |
| Work time missed | 8.63 ±1.26, (6.16,11.11) | 5.13 ±0.90, (3.38,6.89) | 0.014 |
| Impairment while working | 34.25 ±1.52, (31.26,37.23) | 20.98 ±1.06, (18.90,23.07) | <0.001 |
| Overall work impairment | 37.14 ±1.67, (33.88,40.41) | 23.33 ±1.18, (21.03,25.64) | <0.001 |
| Activity impairment | 39.76 ±1.25, (37.32,42.21) | 25.34 ±0.89, (23.60,27.08) | <0.001 |

^a^Adjusted data from multivariate analysis controlling for age, gender, migraine headache day frequency, comorbidities, duration of illness, preventive medication use, with or without aura. ^b^P-values from test of two group difference: t-test for continuous variables and chi-square for categorical variables. ^c^MIDAS disability category data are unadjusted. ^d^Patients completed the EQ-5D 5L; scores were cross-walked to the 3L version [1, 2]. The number of patients with data varied by subgroup size.

EQ-5D=EuroQol 5-Dimensions questionnaire; MIDAS=Migraine Disability Assessment; MSQ=Migraine-Specific Quality of Life Questionnaire; PRO=patient-reported outcome; SD=standard deviation; TIR=triptan insufficient responder; TR=triptan responder; VAS=visual analog scale; WPAI=Work Productivity and Activity Impairment.

## Supplementary references

1. van Reenen M, Janssen B, Stolk E, et al. EuroQol Research Foundation. EQ-5D-5L User Guide, 2019. Available at: https://euroqol.org/publications/user-guides. Accessed March 2020.

2. EuroQoL. NICE position statement on the EQ-5D-5L. Available at https://euroqol.org/nice-position-statement-on-the-eq-5d-5l/. Accessed March 2020.
